# 606. An Evaluation of the Efficacy of Regional Anesthesia in Reducing Donor Site Pain

**DOI:** 10.1093/jbcr/irag033.417

**Published:** 2026-04-06

**Authors:** Lucy Wibbenmeyer, Drew M Danner, Franklin Dexter, Nada Sadek

**Affiliations:** University of Iowa Health Care, Iowa City, IA; University of Iowa Burn Center, West Des Moines, IA; University of Iowa, Iowa City, IA; University of Iowa Health Care, Iowa City, IA

## Abstract

**Introduction:**

Donor site pain remains problematic for burn patients. While pain treatment is multimodal, opioids remain the mainstay. The efficacy and use of regional anesthesia for donor site pain is understudied. The purpose of this study was to assess the impact of donor site blocks on reported pain and post-operative opioid use.

**Methods:**

Patients admitted to the burn center from 2011-2025 who had thigh donor sites were included in the study. Data including demographics, burn history, hospital course, and regional anesthesia procedure were collected from the burn registry and EMR system. Outcome variables included pain ratings and opioid use in mg morphine equivalents (OMEs). Descriptive statistics were used to determine differences between the block and non-block groups, with additional analysis based on regional anesthesia subgroups. Standardized differences (SMD) larger than 0.25 were considered potentially substantive.

**Results:**

The retrospective cohort study included 524 patients, 164 (31.3%) with donor site blocks and 360 (68.7%) without. Overall, the majority were male (394, 75.2%) with an average TBSA of 8.5%, (0.4-72.5). The groups were well-matched in demographics, burn injury, and hospital course data. Overall preoperative (pre-op) pain was 3.8 ± 2.4 and post operative (post-op) pain at 0-24, 0-48 and 0-72 hours was 5.2 ± 2.1, 4.8 ± 1.9, and 4.8 ± 1.9, respectively. The highest OMEs occurred during hours 0-24, doubling from pre-op (51.3 ± 57.3 pre-op v 105.3 ± 80.7 post-op). There was no difference between pain ratings or OME received between the groups. Within the block subgroups, indwelling catheter patients (87, 53.0%) had higher OMEs (71.4 vs 34.94, SMD 0.68) and pain (4.2 vs 3.4, SMD 0.31) in the preoperative period than patients who received single shots (77, 47%). In patients with catheters, groin catheters (30, 34.3%) remained in place for 42.2 ± 15.2 hours and epidurals (57, 65.6%) remained in place for 47.8 ± 24.2 hours, with groin catheters having higher OMEs and reported pain across multiple periods.

**Conclusions:**

This single center, retrospective study shows no substantive difference in subjective pain control or opioid use with donor site regional anesthesia. However, the subjective and non-specific pain ratings, the absence of uniform analgesia protocols, and the inability to control confounding variables limit findings. As regional anesthesia adds to the cost of care, a prospective study is needed to determine its role in donor site pain analgesia.

**Applicability of Research to Practice:**

The findings of this study also highlight the difficulties in assessing subjective values such as pain and support the need for collaboration between burn and anesthesia providers. This study has the potential to lead to standardized protocols for pain-control in the burn unit, substantially improving the experiences of patients.

**Funding for the study:**

Medical School Funding.

